# Location of plant species in Norway gathered as a part of a survey vegetation mapping programme

**DOI:** 10.1016/j.dib.2015.10.014

**Published:** 2015-10-21

**Authors:** Anders Bryn, Hans-Petter Kristoffersen, Michael Angeloff, Ingvild Nystuen, Linda Aune-Lundberg, Dag Endresen, Christian Svindseth, Yngve Rekdal

**Affiliations:** a Norwegian Institute of Bioeconomy Research, POB 115, 1431 Ås, Norway; b Natural History Museum, University of Oslo, POB 1172 Blindern, 0318 Oslo, Norway

**Keywords:** Georeferenced localities, Global Biodiversity Information Facility, Vascular plants, Vegetation plots, Vegetation type

## Abstract

Georeferenced species data have a wide range of applications and are increasingly used for e.g. distribution modelling and climate change studies. As an integrated part of an on-going survey programme for vegetation mapping, plant species have been recorded. The data described in this paper contains 18.521 registrations of plants from 1190 different circular plots throughout Norway. All species localities are georeferenced, the spatial uncertainty is provided, and additional ecological information is reported. The published data has been gathered from 1991 until 2015. The entries contain all higher vascular plants and pteridophytes, and some cryptogams. Other ecological information is also provided for the species locations, such as the vegetation type, the cover of the species and slope. The entire material is stored and available for download through the GBIF server.

**Specifications table**

**Table:** 

Subject area	Biology
More specific subject area	Vegetation ecology and botany
Type of data	List of plant species and location
How data was acquired	Through field-work
Data format	Table
Experimental factors	Uncertain species and locations filtered out
Experimental features	NA
Data source location	Country: Norway
Character set	UTF-8
Data format	Darwin Core Archive, version 1.0 [2]
Dataset identifier	http://doi.org/10.15468/na7jbv[13]
Data accessibility	Data is with this article and accessible for download at GBIF: http://www.gbif.org/dataset/1daaaa9b-f637-4d6a-84d4-d8038d4c71aa

**Value of the data**

• The species locations are gathered within plots, providing co-occurrences of plant species and thus enabling analyses at the community level.
• The recordings are useful for species distribution modelling, since the spatial precision is high and species absences from plots can be derived.
• Many recordings are far away from roads and other infrastructure, thereby providing data from remote areas with few previous recordings.
• Additional ecological information is provided, for example slope and vegetation type, which opens for ecological studies.

## 1 Data

The data described in this paper contain 18.521 georeferenced entries of higher vascular plants and pteridophytes identified in the field. The most common bryophytes and lichens have also been registered, but the registration of these species groups is incomplete and varying strongly. Each entry is linked to a circular plots of 10 m^2^ size, where ecological variables have also been registered (Table 1). The average number of entries in the 1190 plots is 15.6 species. The entries have recently been published (4th October 2015) on the GBIF-server, they are stored there, and are available for download [5].

### 1.1 Temporal coverage

**Fig. 1:**
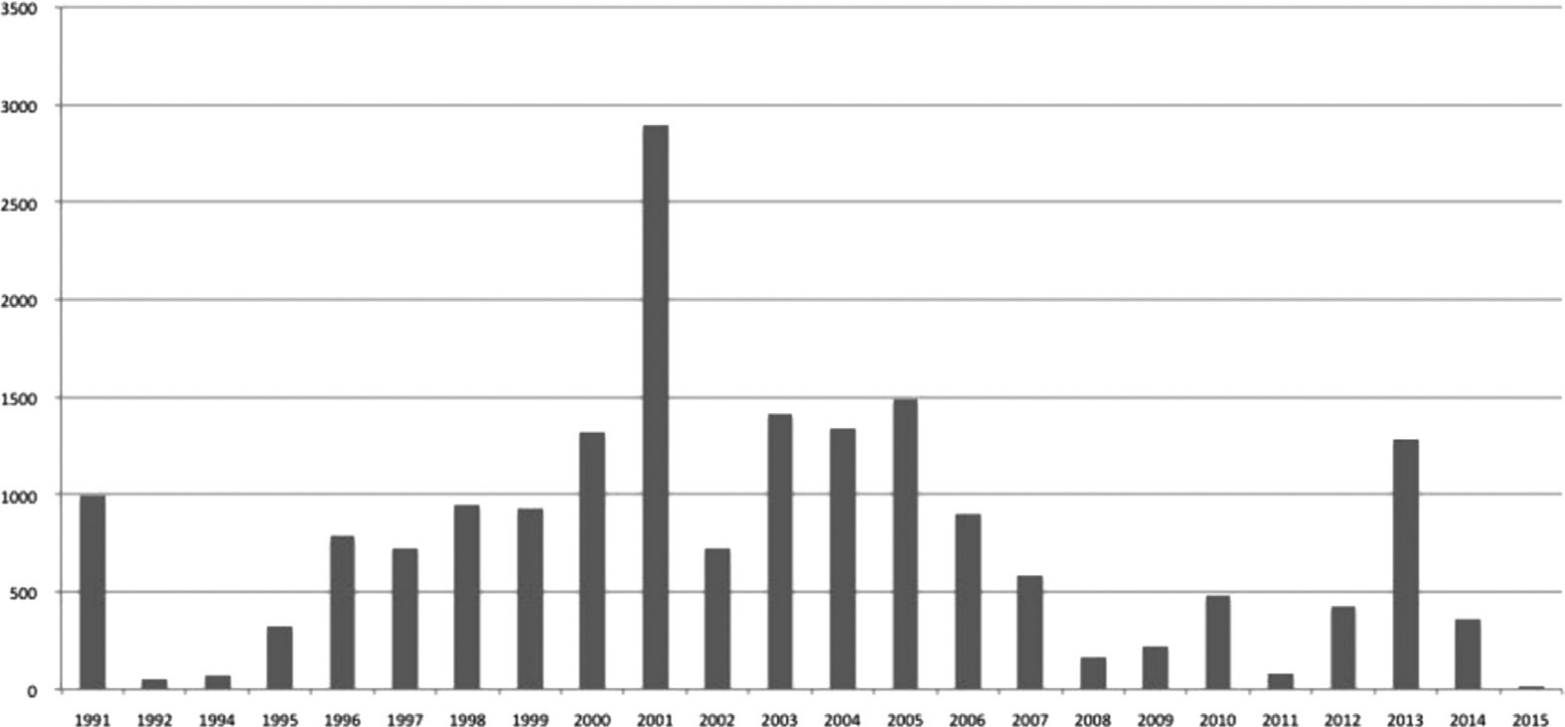
Number of occurrence records per year.

The data has been gathered from 1991 to 2015, varying in number from 2897 entries in the year 2001 to 13 entries in 2015 (Fig. 1). The vegetation survey is still ongoing and new data records from the coming years will be added as they are collected and become available.

### 1.2 Geographic coverage

**Fig. 2:**
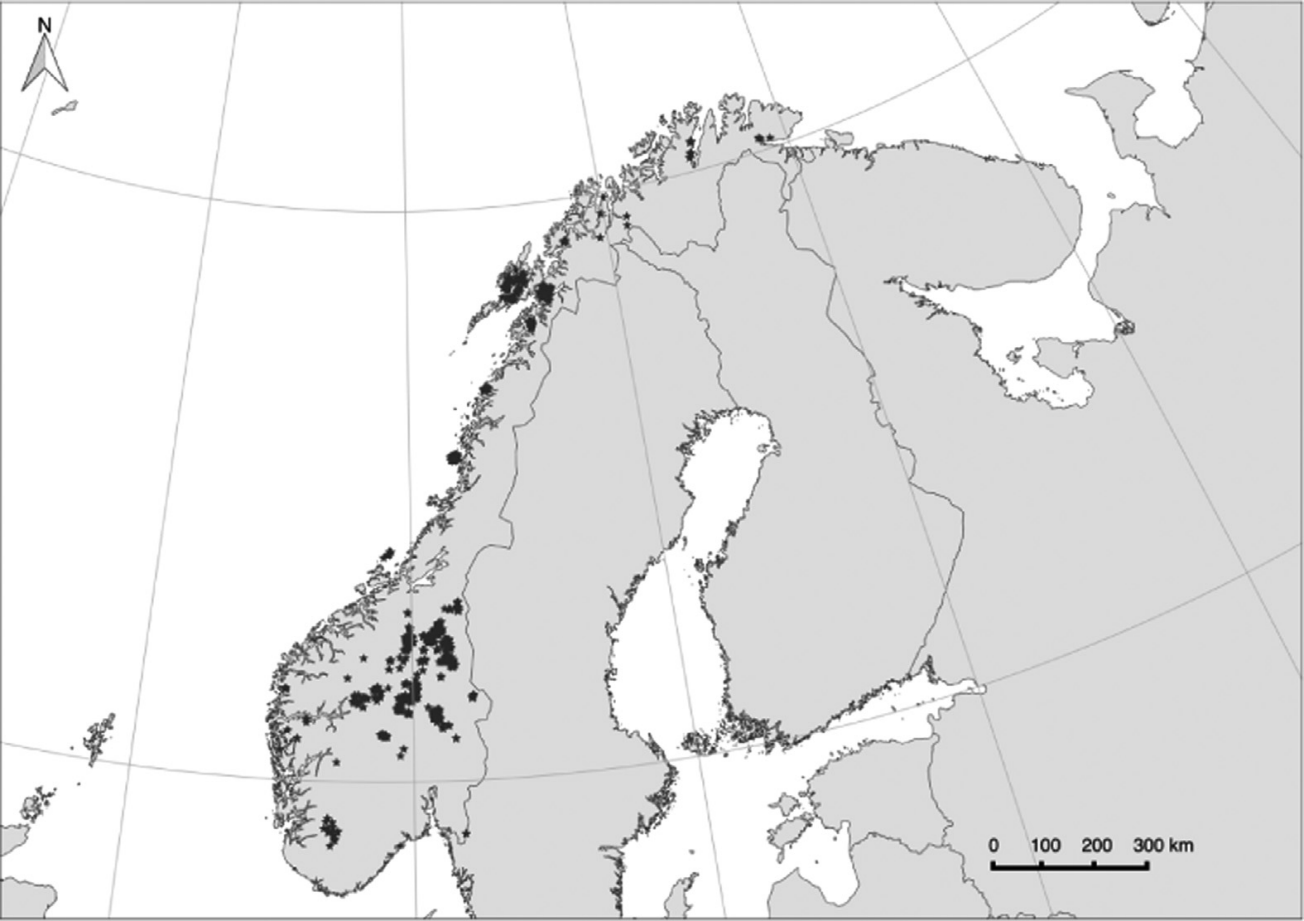
Distribution of the plots in which plant species have been registered.

The species have been sampled from all over Norway, but some regions have far more entries than others (Fig. 2). The dataset is part of a national Norwegian survey vegetation mapping including vegetation observations from 1190 localities (within 53 municipalities) across Norway.

### 1.3 Taxonomic and environmental coverage

This dataset includes occurrence data from 96 families, 254 genera, and 478 species (489 taxa). A list of families with the number of occurrences is provided in Appendix A. The data has been gathered within the boreal and alpine bioclimatic regions [1].

## 2 Experimental design, materials and methods

### 2.1 Background

The data has been registered as a part of a national Norwegian survey vegetation mapping programme [2], [10], and all species localities are therefore within areas documented through vegetation maps. The vegetation type system used for mapping has been almost stable during the entire sampling period, from 1991 until 2015 [8]. The vegetation type system used for mapping has 45 vegetation types and 9 other land cover types, and is adapted to a mapping scale of 1:20.000–1:50.000 [9]. To enable sufficient descriptions of the vegetation types, and how they vary regionally and throughout Norway, species has been registered systematically as a part of the mapping programme.

### 2.2 The species were registered within circular plots

Within each area targeted for mapping in the national Norwegian survey vegetation mapping programme, representative locations of the vegetation types have been documented by recording plant species within circular plots. A total number of 1190 plots are surveyed. All entries uploaded in the GBIF database are therefore linked to one of these circular plots. More than 99.6% (18.460 entries) of the species recorded have been located within plots of 10 m^2^ (radius c. 1.8 m). The size of the plot is provided for all recordings (Table 1).

### 2.3 Information linked to each species registration and how they were measured

The data provided follow the standard given by GBIF, i.e. the Darwin Core data standard [11], [12] and the Darwin Core Archive formatting rules [3]. Alongside the sampling of species within each plot, additional ecological information has been registered for the plot (Table 1). All the information provided has been gathered during field-work by 11 different surveyors between May and October each summer.

Since 2003, all plots have been georeferenced using GPS, giving an estimated average uncertainty of ±5 m. From 1991 until 2003, the plots were georeferenced using topographic maps, resulting in an estimated average uncertainty of ±100 m.

The species cover represents each species׳ total vertical cover of the entire 10 m^2^ plot. Trees do not have to be rooted inside the plot, but must vertically cover the plot. Recording of bush cover, field-layer cover and so forth follow the same logic, except for the tree cover. The tree cover has been recorded for a larger circular plot of 25 m^2^ (including the original 10 m^2^ plot). The cover has been registered in two ways; as cover within Hult-Sernanders׳ 6 classes [6], or/and as percentage cover. Hult-Sernander has been registered for 99.6% of all entries, whereas percentage cover has been registered for 66.3% of all entries. 23 entries lack a measure of the cover. Aspect and slope has been recorded for each plot, representing the mean aspect and slope of each 10 m^2^ plot.

### 2.4 Quality control

All records have been validated before they were exported to GBIF. The 18.521 species entries that have been published, makes up 84 percentage of the original database. 3542 species entries were erased based on various errors. The most common errors were missing coordinates, uncertain identification of the species, or incomplete taxonomy (only genus provided). All such records were erased. In addition, we erased all plots with unlikely geographical coordinates, for example plots located outside of the original mapping area of the project they belonged to.

Difficult or doubtful species or individuals were originally registered, but have been erased from the data as part of the quality control. All species names were corrected according to the Species Nomenclature Database, provided by the Norwegian Biodiversity Information Centre [7]. During that process, the higher nomenclature for each species was added to all records, from Kingdom to Taxon.

## 3 Usage rights

Data is published under the Creative Commons Zero (CC0) public domain waiver [14], [15]. The data in the dataset is published free and open for scientific reuse and other legible use. Please cite this publication or the resource when using parts of the data in your analyses.

## Figures and Tables

**Table 1:** Additional information gathered for each plot and represented for each 18.521 species entry. When downloading data from GBIF, the information will be arranged according to the Darwin Core 2 data standard [3], but including the information provided in this table.

Type of information	Data type	Scale

Species name	Categorical	Latin and Norwegian names
Taxonomy	Categorical	Provided by NBIC [7]
Coordinates	−	Longitude and Latitude
Coordinate precision	Ordinal	± metres
Altitude	Ordinal	Metre above sea level
Date	−	Day, month, year
Name of registrar	Categorical	Full name provided
Size of the plot	Categorical	m^2^
Locality name	−	According to topographic maps
Municipality name	−	According to topographic maps
Survey vegetation types	Categorical	54 vegetation types [9]
Detailed vegetation types	Categorical	137 vegetation types [4]
Aspect	Categorical	0–8 (N, NE, E, SE etc)
Slope	Ordinal	0–90
Species cover	Ordinal	0–100 (%) or 0–5 (Hult-Sernander)
Tree cover	Ordinal	0–100 (%) or 0–5 (Hult-Sernander)
Bush cover	Ordinal	0–100 (%) or 0–5 (Hult-Sernander)
Field-layer cover	Ordinal	0–100 (%) or 0–5 (Hult-Sernander)
Bottom-layer cover	Ordinal	0–100 (%) or 0–5 (Hult-Sernander)
Cover of dead litter	Ordinal	0–100 (%) or 0–5 (Hult-Sernander)
Cover of bare rocks and gravel	Ordinal	0–100 (%) or 0–5 (Hult-Sernander)

## Footnotes

Appendix A Supplementary data associated with this article can be found in the online version at doi:10.1016/j.dib.2015.10.014.
