# Supplementary material for: Location of plant species in Norway gathered as a part of a survey vegetation mapping programme
Source: Data Brief. 2015 Oct 21;5:589–94. doi: 10.1016/j.dib.2015.10.014 (PMC4773408; doi:10.1016/j.dib.2015.10.014)
Supplement: Supplementary file 1 — Supplementary material [file mmc1.doc]

**Appendix A**

Number of occurrences from plant families. Families are given in alphabetical order.

| Plant families | # of occurrences |
| --- | --- |
| Adiantaceae | 6 |
| Adoxaceae | 1 |
| Amaranthaceae | 1 |
| Amaryllidaceae | 1 |
| Amblystegiaceae | 11 |
| Antheliaceae | 1 |
| Apiaceae | 61 |
| Asparagaceae | 80 |
| Aspleniaceae | 1 |
| Asteraceae | 931 |
| Asterales | 2 |
| Aulacomniaceae | 1 |
| Balsaminaceae | 1 |
| Betulaceae | 784 |
| Blechnaceae | 14 |
| Boraginaceae | 28 |
| Brassicaceae | 27 |
| Campanulaceae | 118 |
| Caprifoliaceae | 119 |
| Caryophyllaceae | 237 |
| Caryophyllales | 1 |
| Celastraceae | 55 |
| Cladoniaceae | 602 |
| Climaciaceae | 14 |
| Cornaceae | 168 |
| Crassulaceae | 37 |
| Cupressaceae | 363 |
| Cyperaceae | 1505 |
| Dennstaedtiaceae | 7 |
| Diapensiaceae | 6 |
| Dicranaceae | 2 |
| Droseraceae | 27 |
| Dryopteridaceae | 64 |
| Equisetaceae | 169 |
| Ericaceae | 2577 |
| Fabaceae | 206 |
| Fagaceae | 2 |
| Gentianaceae | 19 |
| Geraniaceae | 271 |
| Grimmiaceae | 28 |
| Grossulariaceae | 8 |
| Hylocomiaceae | 534 |
| Hypericaceae | 5 |
| Icmadophilaceae | 4 |
| Juncaceae | 541 |
| Juncaginaceae | 5 |
| Lamiaceae | 36 |
| Lentibulariaceae | 70 |
| Liliaceae | 1 |
| Linaceae | 7 |
| Lophoziaceae | 20 |
| Lycopodiaceae | 153 |
| Malpighiales | 2 |
| Meesiaceae | 4 |
| Melanthiaceae | 28 |
| Menyanthaceae | 31 |
| Montiaceae | 1 |
| Myricaceae | 10 |
| Nartheciaceae | 27 |
| Nephromataceae | 4 |
| Onagraceae | 53 |
| Ophioglossaceae | 12 |
| Orchidaceae | 99 |
| Orobanchaceae | 379 |
| Oxalidaceae | 91 |
| Parmeliaceae | 339 |
| Peltigeraceae | 40 |
| Pertusariaceae | 3 |
| Pinaceae | 197 |
| Plagiotheciaceae | 3 |
| Plantaginaceae | 133 |
| Poaceae | 2709 |
| Poales | 5 |
| Polemoniaceae | 1 |
| Polygalaceae | 8 |
| Polygonaceae | 567 |
| Polypodiaceae | 2 |
| Primulaceae | 375 |
| Ptilidiaceae | 13 |
| Ranunculaceae | 560 |
| Ranunculales | 2 |
| Rhamnaceae | 1 |
| Rosaceae | 1188 |
| Rosales | 3 |
| Rubiaceae | 97 |
| Salicaceae | 716 |
| Saxifragaceae | 112 |
| Selaginellaceae | 63 |
| Sphaerophoraceae | 6 |
| Sphagnaceae | 16 |
| Tamaricaceae | 1 |
| Thelypteridaceae | 83 |
| Tofieldiaceae | 39 |
| Urticaceae | 8 |
| Violaceae | 307 |
| Woodsiaceae | 251 |
